# Graphene wrapped ordered LiNi_0.5_Mn_1.5_O_4_ nanorods as promising cathode material for lithium-ion batteries

**DOI:** 10.1038/srep11958

**Published:** 2015-07-07

**Authors:** Xiao Tang, S. Savut Jan, Yanyan Qian, Hui Xia, Jiangfeng Ni, Serguei V. Savilov, Serguei M. Aldoshin

**Affiliations:** 1 School of Materials Science and Engineering, Nanjing University of Science and Technology, Nanjing 210094, China; 2 Herbert Gleiter Institute of Nanoscience, Nanjing University of Science and Technology, Nanjing 210094, China; 3 College of Physics, Optoelectronics and Energy, Soochow University, Suzhou 215006, China; 4 Department of Chemistry, M. V. Lomonosov Moscow State University, Moscow 119991, Russia; 5 Department of Physical Chemistry Engineering, M. V. Lomonosov Moscow State University, Moscow 119991, Russia

## Abstract

LiNi_0.5_Mn_1.5_O_4_ nanorods wrapped with graphene nanosheets have been prepared and investigated as high energy and high power cathode material for lithium-ion batteries. The structural characterization by X-ray diffraction, Raman spectroscopy, and Fourier transform infrared spectroscopy indicates the LiNi_0.5_Mn_1.5_O_4_ nanorods prepared from β-MnO_2_ nanowires have ordered spinel structure with *P*4_3_32 space group. The morphological characterization by scanning electron microscopy and transmission electron microscopy reveals that the LiNi_0.5_Mn_1.5_O_4_ nanorods of 100–200 nm in diameter are well dispersed and wrapped in the graphene nanosheets for the composite. Benefiting from the highly conductive matrix provided by graphene nanosheets and one-dimensional nanostructure of the ordered spinel, the composite electrode exhibits superior rate capability and cycling stability. As a result, the LiNi_0.5_Mn_1.5_O_4_-graphene composite electrode delivers reversible capacities of 127.6 and 80.8 mAh g^−1^ at 0.1 and 10 C, respectively, and shows 94% capacity retention after 200 cycles at 1 C, greatly outperforming the bare LiNi_0.5_Mn_1.5_O_4_ nanorod cathode. The outstanding performance of the LiNi_0.5_Mn_1.5_O_4_-graphene composite makes it promising as cathode material for developing high energy and high power lithium-ion batteries.

Renewable and sustainable energy resources, such as solar, wind, and tide, attract more and more attention because of the shortage of fossil fuels and their associated environmental problems. However, these renewable energy resources are only intermittently available and require energy storage systems to improve the power reliability and quality^1^,^2^. Lithium-ion batteries with high energy density, light weight, long cycle life, and environmental friendliness have recently shown potential applications in the areas of electric vehicles and stationary energy storage for smart grids^3^. However, higher requirements are raised by these applications, and further improvements in terms of energy and power densities, safety, and lifetime are imperative for lithium-ion batteries^4^.

To further increase the energy density for lithium-ion batteries, various positive electrode materials have been widely investigated to obtain either high working voltage or large capacity. Among them, LiNi_0.5_Mn_1.5_O_4_ spinel is particularly attractive due to its high average discharge voltage of about 4.7 V (vs. Li/Li^+^) with a theoretical capacity of about 148 mAh g^−1^^5^,^6^. LiNi_0.5_Mn_1.5_O_4_ can exist in two different crystallographic structures. The disordered LiNi_0.5_Mn_1.5_O_4-δ_ has the normal face-centered spinel structure with *F*d-3m symmetry, while Mn^4+^ and Ni^2+^ ions order in stoichiometric LiNi_0.5_Mn_1.5_O_4_ resulting in the *P*4_3_32 symmetry^7^,^8^. The disordered spinel shows higher electronic conductivity due to the presence of Mn^3+^/Mn^4+^ redox couple and Ni/Mn disordering^9^. However, the nonstoichiometry in disordered spinel often induces impurities such as NiO and Li_x_Ni_y_O, and the 4 V (vs. Li/Li^+^) Mn^3+^/Mn^4+^ redox couple, thus reducing the specific capacity and energy^10^. It is still a challenge to achieve both high energy and high power densities for the ordered spinel due to its poor electronic conductivity. Moreover, the high working voltage at about 4.7 V (vs. Li/Li^+^) induces side reactions at the electrode/electrolyte interface, resulting in continuous capacity fading during the cycling^11^.

To circumvent the drawbacks of the high voltage spinels, two strategies are often employed to improve the electrochemical performance. One approach is to construct nanostructures for the spinel, which can drastically shorten the transport distance for both electrons and lithium ions, resulting in greatly improved rate capability^12^,^13^,^14^,^15^. However, the increased surface area associated with nanostructuring will aggravate the side reactions at high voltage and deteriorate the capacity fading during cycling. Another approach people often used is to modify the spinel surface by coating a thin protective layer, which can greatly improve the interface stability with enhanced cycling performance^16^,^17^,^18^,^19^,^20^. However, the coating materials, most of which are metal oxides and metal fluorides, only function as protective layers for the spinel without improvement in electrical conductivity. Therefore, new strategies that can combine the advantages of the above mentioned two approaches need to be developed to further improve the electrochemical performance of the high voltage spinels.

Herein, we developed a facile method to prepare graphene nanosheets wrapped ordered LiNi_0.5_Mn_1.5_O_4_ nanorods as high energy and high power cathode material for lithium-ion batteries (Fig. 1). In the hybrid electrode design, the one-dimensional nanostructure of LiNi_0.5_Mn_1.5_O_4_ enables fast lithium ion transport while the graphene wrapping suppresses the side reactions at high voltage and further improves the electron transfer. It has been demonstrated that the graphene or grahene oxide nanosheets incorporation can greatly improve the rate capability and cycling stability for the disordered spinels^21^,^22^. Consequently, the ordered LiNi_0.5_Mn_1.5_O_4_ nanorods-graphene composite cathode exhibited greatly improved cycling performance and rate performance compared to the bare ordered LiNi_0.5_Mn_1.5_O_4_ nanorods. The promising results indicate the great potential of developing high energy and high power lithium-ion batteries by utilizing the graphene nanosheets wrapped ordered LiNi_0.5_Mn_1.5_O_4_ nanorods.

## Results

**Figure 2:**
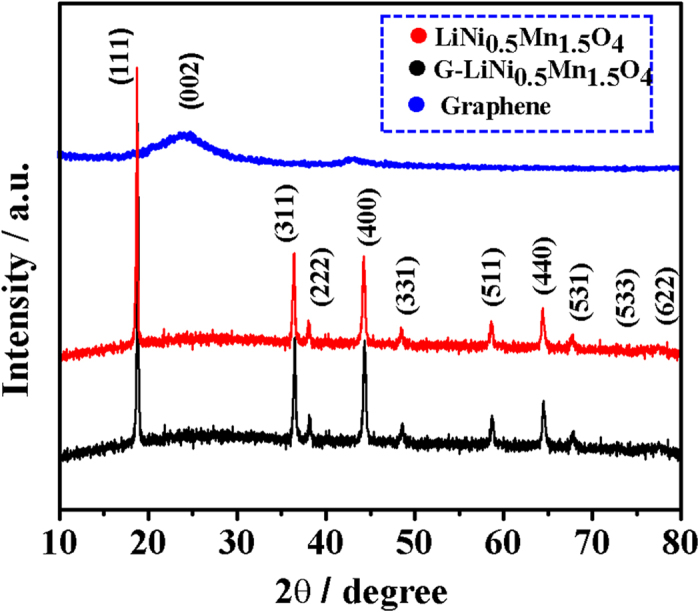
XRD patterns of the pristine graphene nanosheets, the as-synthesized LiNi_0.5_Mn_1.5_O_4_ nanorods, and the LiNi_0.5_Mn_1.5_O_4_-graphene composite.

The hydrothermally prepared β-MnO_2_ nanowires were used as the template and the XRD pattern shows no trace of impurity (Fig. S1, Supporting Information). Figure 2 shows the XRD patterns of the pristine graphene nanosheets, the as-synthesized LiNi_0.5_Mn_1.5_O_4_ nanorods, and the LiNi_0.5_Mn_1.5_O_4_-graphene composite. The XRD pattern of the graphene shows a small hump at about 26°, which can be attributed to the (002) reflection of graphite. The as-synthesized LiNi_0.5_Mn_1.5_O_4_ nanorods and the LiNi_0.5_Mn_1.5_O_4_-graphene composite show similar XRD patterns, which can be indexed to the cubic spinel structure with space group *P*4_3_32 (JCPDS No. 80-2184). No impurity peaks from NiO or Li_x_Ni_y_O can be detected, indicating the existence of pure spinel phase. Rietveld refinement gives a lattice parameter of *a* = 8.169 Å, which agrees well with reported value for the ordered LiNi_0.5_Mn_1.5_O_4_^12^,^13^. No diffraction peaks of graphene can be observed from the XRD pattern of the LiNi_0.5_Mn_1.5_O_4_-graphene composite, which is probably due to the strong diffraction peaks from the highly crystalline LiNi_0.5_Mn_1.5_O_4_ and the nanoscale size feature of low content graphene. However, the superstructure peaks, which are characteristics of Ni and Mn ordering, cannot be resolved from the XRD patterns of the spinels because of their low intensities^23^. Therefore, further structural investigation by Raman and FTIR are required to confirm the *P*4_3_32 symmetry of the synthesized spinel in this work.

**Figure 3:**
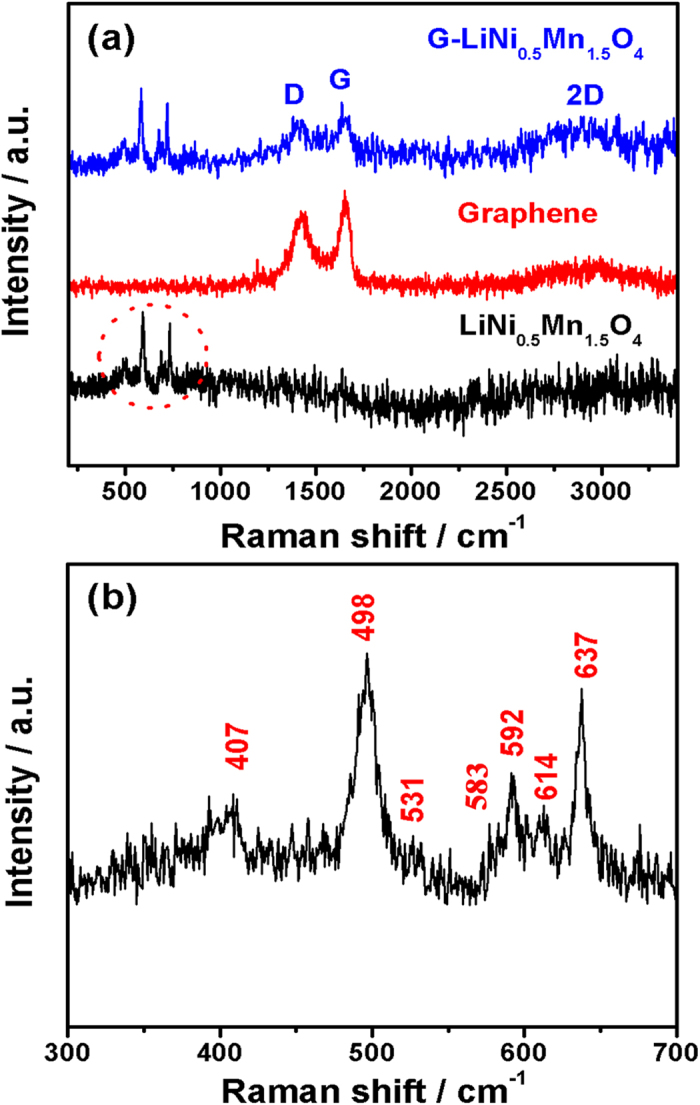
(**a**) Raman spectra of the pristine graphene nanosheets, the bare LiNi_0.5_Mn_1.5_O_4_ nanorods, and the LiNi_0.5_Mn_1.5_O_4_-graphene composite. (**b**) Enlargement of the Raman spectrum of the LiNi_0.5_Mn_1.5_O_4_ nanorods in the frequency range between 300 and 700 cm^−1^.

Raman spectra of the pristine graphene nanosheets, the bare LiNi_0.5_Mn_1.5_O_4_ nanorods, and the LiNi_0.5_Mn_1.5_O_4_-graphene composite are shown in Fig. 3a. The Raman spectra of the bare LiNi_0.5_Mn_1.5_O_4_ nanorods and the LiNi_0.5_Mn_1.5_O_4_-graphene composite show similar features in the frequency range of 300–1000 cm^−1^, which are in good agreement with published works on ordered LiNi_0.5_Mn_1.5_O_4_ spinel^23^,^24^. The Raman spectrum of the LiNi_0.5_Mn_1.5_O_4_ nanorods in the frequency range between 300 to 700 cm^−1^ was enlarged and shown in Fig. 3b, revealing six Raman bands. The strong band around 638 cm^−1^ is assigned to the symmetric Mn-O stretching mode of MnO_6_ octahedral (A_1g_), while the two bands at 407 and 498 cm^−1^ are associated with the Ni^2+^-O stretching mode in the structure^25^. The peak near 580–620 cm^−1^ is considered as T2g^(3)^ of the spinel compound, and the split of T2g^(3)^ is the strong evidence of the ordered spinel due to its low symmetry (*P*4_3_32). In the frequency range between 1000 and 3700 cm^−1^, the Raman spectrum of the LiNi_0.5_Mn_1.5_O_4_-graphene composite show similar Raman features as the pristine graphene nanosheets, revealing the characteristic D band, G band, and 2D band of graphene.

**Figure 4:**
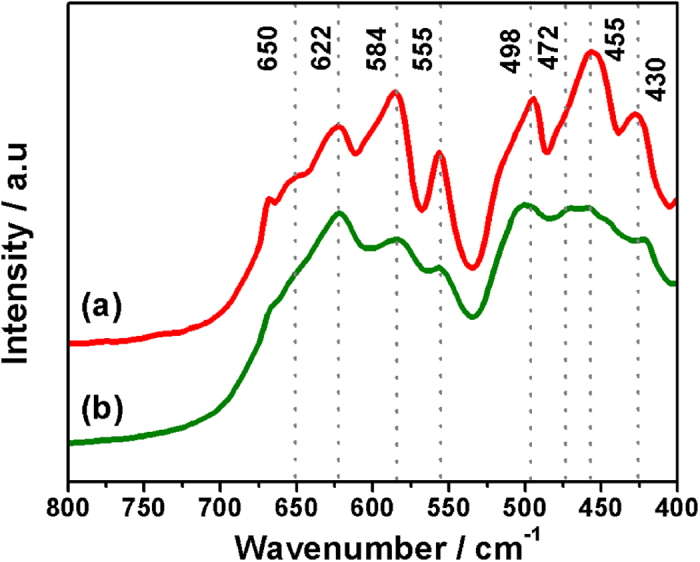
FTIR spectra of(**a**) the ordered LiNi_0.5_Mn_1.5_O_4_ nanorods and (**b**) the disordered LiNi_0.5_Mn_1.5_O_4_ powder prepared by a solid state synthesis.

To further confirm the structural symmetry of the spinel in the present work, FTIR analysis was carried out on different spinel powder samples. For comparison, disordered LiNi_0.5_Mn_1.5_O_4_ powders were prepared by a solid state synthesis according to the literature^26^. Figure 4a,b show the FTIR spectra of the as-prepared LiNi_0.5_Mn_1.5_O_4_ nanorods and the LiNi_0.5_Mn_1.5_O_4_ powders prepared by solid state synthesis, respectively. There are eight distinctive bands for the as-prepared LiNi_0.5_Mn_1.5_O_4_ nanorods, and the observed wavenumbers match well with those of ordered spinel in literature^8^,^23^. By contrast, some bands are not well resolved for the disordered LiNi_0.5_Mn_1.5_O_4_ because of lacking of Ni/Mn ordering. Another evidence to distinguish the two structures is the change of the intensity ratio of the two bands at 622 and 584 cm^−1^. In specific, the 584 cm^−1^ Ni-O band increases in intensity compared to the 622 cm^−1^ Mn-O band with increasing level of ordering^8^. Agreeing well with Raman analysis, the FTIR analysis confirms that the as-prepared LiNi_0.5_Mn_1.5_O_4_ nanorods have ordered spinel structure with *P*4_3_32 symmetry.

**Figure 5:**
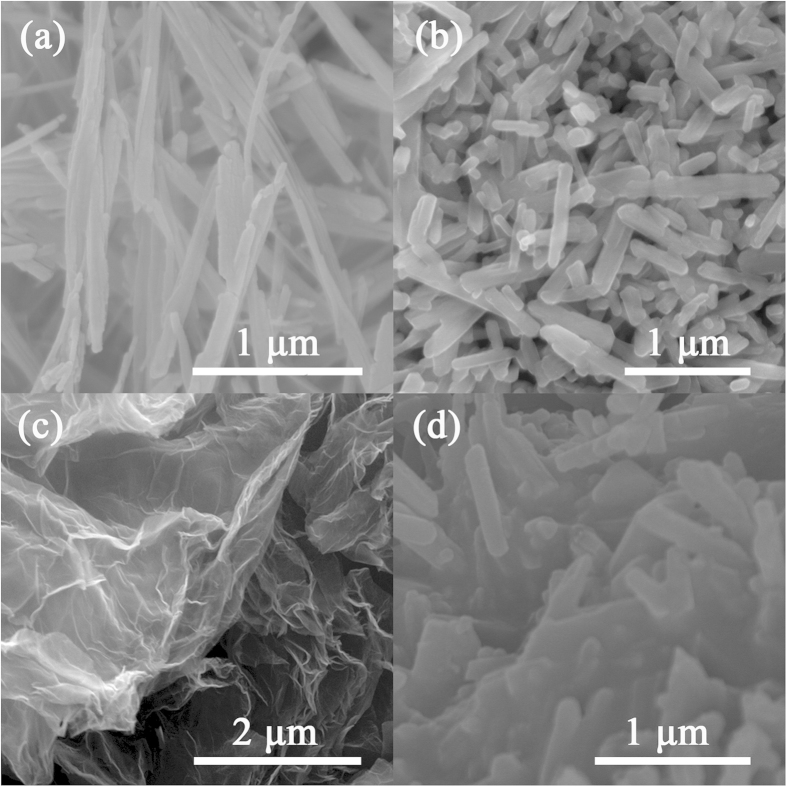
FESEM images of(**a**) the β-MnO_2_ nanowires, (**b**) the LiNi_0.5_Mn_1.5_O_4_ nanorods, (**c**) the pristine graphene nanosheets, and (**d**) the LiNi_0.5_Mn_1.5_O_4_-graphene composite.

The typical morphologies of β-MnO_2_ nanowires, LiNi_0.5_Mn_1.5_O_4_ nanorods, graphene nanosheets, and LiNi_0.5_Mn_1.5_O_4_-graphene composite are shown in Fig. 5. As shown in Fig. 5a, the β-MnO_2_ nanowires are about 50–100 nm in diameter and 2-3 μm in length. Figure 5b reveals that the as-synthesized LiNi_0.5_Mn_1.5_O_4_ nanorods well preserve the one-dimensional morphology of the β-MnO_2_ nanowires. However, the LiNi_0.5_Mn_1.5_O_4_ nanorods have larger diameters of about 100–200 nm and shorter lengths of about 0.5–1 μm, which are probably caused by the phase transition and breaking of β-MnO_2_ nanowires due to the strain associated with the volume expansion. As shown in Fig. 5c, the prepared graphene nanosheets are corrugated and transparent, well resembling the two-dimensional morphology. LiNi_0.5_Mn_1.5_O_4_-graphene composite shows a laminated morphology (Fig. 5d) with LiNi_0.5_Mn_1.5_O_4_ nanorods well dispersed on the graphene nanosheets without severe aggregation.

**Figure 6:**
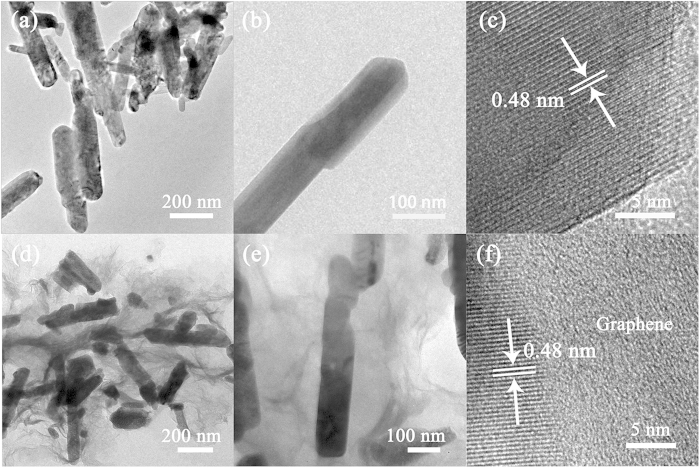
(**a**–**c**) TEM and HRTEM images of the bare LiNi_0.5_Mn_1.5_O_4_ nanorods. (**d**–**f**) TEM and HRTEM images of the LiNi_0.5_Mn_1.5_O_4_-graphene composite.

Figure 6a,b show the TEM images of the LiNi_0.5_Mn_1.5_O_4_ nanorods with low and high magnifications, respectively, revealing the single crystalline feature of the nanorods. The interplanar spacing determined by the HRTEM image in Fig. 6c is about 0.48 nm, which is consistent with the (111) planes of LiNi_0.5_Mn_1.5_O_4_ spinels. For the LiNi_0.5_Mn_1.5_O_4_-graphene composite, Fig. 6d,e clearly reveal that the LiNi_0.5_Mn_1.5_O_4_ nanorods are well distributed and wrapped in the transparent graphene nanosheets. The HRTEM image in Fig. 6f shows a clear interface between one LiNi_0.5_Mn_1.5_O_4_ nanorod and one graphene sheet, indicating good adhesion and close contact between the two components in the composite. The interplanar spacing of about 0.48 nm shown in Fig. 6f is also attributed to the (111) planes of the spinel. The uniform LiNi_0.5_Mn_1.5_O_4_-graphene heterostructure can be attributed to the facile chemical mixing method. Initially, the mortar grinding can effectively separate the LiNi_0.5_Mn_1.5_O_4_ nanorods from aggregation. Afterward, the ultrasonication and the continuous stirring facilitate the uniform nanorods distribution in the graphene matrix, resulting the graphene wrapped LiNi_0.5_Mn_1.5_O_4_ nanorods. The graphene content was determined to be about 4.7 wt% by TGA measurement (Fig. S2, Supporting Information).

**Figure 7:**
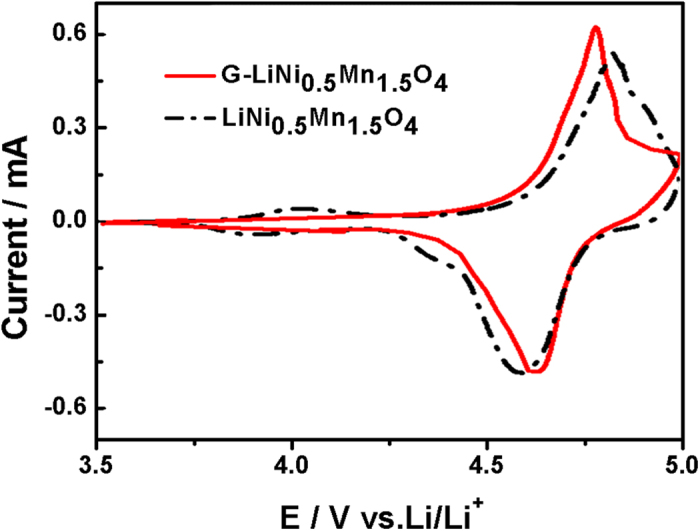
Typical CV curves of the bare LiNi_0.5_Mn_1.5_O_4_ nanorod electrode and the LiNi_0.5_Mn_1.5_O_4_-graphene composite electrode.

To evaluate the electrochemical properties of the LiNi_0.5_Mn_1.5_O_4_-graphene composite, half cells were assembled using lithium foil as both counter and reference electrodes. Fig. 7 compares the typical CV curves of the bare LiNi_0.5_Mn_1.5_O_4_ nanorod electrode and the LiNi_0.5_Mn_1.5_O_4_-graphene composite electrode between 3.5 and 5 V (vs. Li/Li^+^) at a scan rate of 0.05 mV s^−1^. For both electrodes, a pair of strong redox peaks at about 4.7 V (vs. Li/Li^+^) can be clearly observed, which can be attributed to the Ni^2+^/Ni^4+^ redox reactions^8^,^27^. The 4 V redox peaks, corresponding to the Mn^3+^/Mn^4+^ redox couple, are almost negligible in the CV curves for the bare LiNi_0.5_Mn_1.5_O_4_ nanorod electrode and the LiNi_0.5_Mn_1.5_O_4_-graphene composite electrode, indicating their nearly perfect stoichiometry. By contrast, apart from the obvious 4 V (vs. Li/Li^+^) redox peaks, the disordered LiNi_0.5_Mn_1.5_O_4_ will show clear two pairs of redox peaks at about 4.7 V (vs. Li/Li^+^) in CV^28^. Previous studies indicate that the redox peak splitting at about 4.7 V (vs. Li/Li^+^) for the high voltage Li_x_Ni_0.5_Mn_1.5_O_4_ (0 < x < 1) is probably due to two separate redox couples of Ni^2+^/Ni^3+^ and Ni^3+^/Ni^4+^ and/or Li/vacancy ordering at x = 0.5^29^. However, such peak splitting is not obvious for the ordered spinel as only one pair of redox peaks are observed in the typical CV curve. As discussed in our previous work on disordered LiNi_0.5_Mn_1.5_O_4-δ_ thin films^30^, the ordering arrangement of Ni and Mn in the ordered spinel is probably not commensurate with the preferred Li/vacancy ordering at x = 0.5 so that the Ni/Mn ordering suppresses Li/vacancy ordering and redox peak splitting at about 4.7 V (vs. Li/Li^+^). In comparison with the bare LiNi_0.5_Mn_1.5_O_4_ nanorod electrode, the LiNi_0.5_Mn_1.5_O_4_-graphene composite electrode shows much smaller peak separation between the cationic peak and anodic peak in the CV curve, indicating the electrode polarization can be greatly reduced by incorporating the graphene nanosheets into LiNi_0.5_Mn_1.5_O_4_ nanorods.

**Figure 8:**
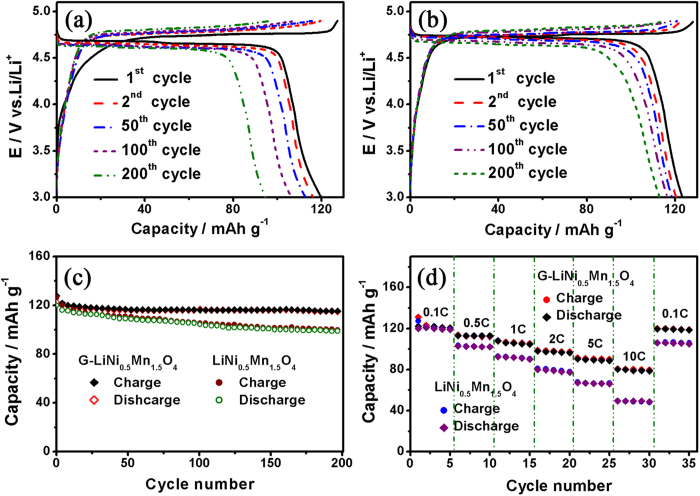
(**a**) Charge/discharge curves of the bare LiNi_0.5_Mn_1.5_O_4_ nanorod electrode at various cycle numbers at 0.1 C rate. (**b**) Charge/discharge curves of the LiNi_0.5_Mn_1.5_O_4_-graphene composite electrode at various cycle numbers at 0.1 C rate. (**c**) Comparison of cycle performance between the bare LiNi_0.5_Mn_1.5_O_4_ nanorod electrode and the LiNi_0.5_Mn_1.5_O_4_-graphene composite electrode. (**d**) Comparison of rate capability between the bare LiNi_0.5_Mn_1.5_O_4_ nanorod electrode and the LiNi_0.5_Mn_1.5_O_4_-graphene composite electrode.

Figure 8a,b show the charge/discharge curves of the bare LiNi_0.5_Mn_1.5_O_4_ nanorod and the LiNi_0.5_Mn_1.5_O_4_-graphene composite electrodes, respectively, at the 1^st^, 2^nd^, 50^th^, 100^th^, and 200^th^ cycles at a current rate of 0.1 C between 3.0 and 4.9 V (vs. Li/Li^+^). Agreeing well with the CV results, the charge/discharge curves of the two electrodes clearly show only one flat voltage plateau around 4.70 V (vs. Li/Li^+^) due to the Ni^2+^/Ni^4+^ redox couple, which is the characteristic electrochemical behavior of the ordered spinel^8^. In comparison, the charge/discharge curves of the LiNi_0.5_Mn_1.5_O_4_-graphene composite electrode show much smaller voltage difference between charge and discharge voltage plateaus, indicating smaller polarization and internal resistance of the spinel electrode with graphene incorporation. The first charge and discharge capacities of the LiNi_0.5_Mn_1.5_O_4_-graphene composite electrode are 127.6 and 122.4 mAh g^−1^, with a coulombic efficiency of about 96%. By contrast, the first charge and discharge capacities of the bare LiNi_0.5_Mn_1.5_O_4_ nanorod electrode are 127.3 and 119.7 mAh g^−1^, respectively, with a coulombic efficiency of about 94%. It is clear that the LiNi_0.5_Mn_1.5_O_4_-graphene composite electrode can deliver a larger reversible capacity and higher coulombic efficiency compared to the bare LiNi_0.5_Mn_1.5_O_4_ nanorod electrode. The larger reversible capacity of the LiNi_0.5_Mn_1.5_O_4_-graphene composite electrode can be attributed to the smaller polarization of the electrode, which favors fast charge transport and increases the utilization of the active material. The initial irreversible capacity loss is partially contributed by the solid electrolyte interface (SEI) layer formation due to the electrolyte decomposition at high voltage^31^. The wrapping with graphene could greatly suppress the SEI layer formation at high voltage, thus improving the initial coulombic efficiency of the composite electrode. Figure 8c compares the cycle performance of the two electrodes, revealing greatly improved cycling stability for the LiNi_0.5_Mn_1.5_O_4_-graphene composite electrode. After 200 cycles at 0.1 C rate, LiNi_0.5_Mn_1.5_O_4_-graphene composite electrode can still deliver a reversible capacity of about 115 mAh g^−1^, retaining 94% of its initial reversible capacity. In comparison, the bare LiNi_0.5_Mn_1.5_O_4_ nanorod electrode only retained 82% of its initial reversible capacity. The capacity fading of the high voltage spinel during cycling is mainly contributed by the structural deterioration induced by Mn^3+^ ion dissolution and internal resistance increase induced by the side reactions at the electrode surface at high voltage^32^,^33^. For the ordered spinel, Mn^3+^ ion dissolution may not be the major reason that causes the capacity fading since there are negligible Mn^3+^ ions in ordered spinel due to its nearly perfect stoichiometry. The side reactions, including SEI layer formation, could be more detrimental to the cycle performance because the increased polarization induced by the increasing resistance will lead to less reversible capacity. As shown in Fig. 8a, the voltage difference between charge and discharge keeps increasing with the cycling test, revealing a obvious cell polarization growth for the bare LiNi_0.5_Mn_1.5_O_4_ nanorod electrode. By contrast, the polarization growth for the LiNi_0.5_Mn_1.5_O_4_-graphene composite electrode is greatly mitigated, which can be attributed to the graphene protection, suppressing the side reactions at the electrode surface. Figure 8d compares the rate capability of the two electrodes by plotting the specific capacity as a function of cycle number at different current rates. The typical charge/discharge curves of bare LiNi_0.5_Mn_1.5_O_4_ nanorod electrode and the LiNi_0.5_Mn_1.5_O_4_-graphene composite electrode at different current rates are shown in Fig. S3 (Supporting Information). It is obvious that the LiNi_0.5_Mn_1.5_O_4_-graphene composite electrode possesses much better rate capability as it can retain more reversible capacity as the discharge rate increases. Even at 10 C rate, the LiNi_0.5_Mn_1.5_O_4_-graphene composite electrode can still deliver a reversible capacity of about 80.8 mAh g^−1^, which is much larger than that of the bare LiNi_0.5_Mn_1.5_O_4_ nanorod electrode (49.2 mAh g^−1^). When the current rate was set back to 0.1 C, the charge and discharge capacities of LiNi_0.5_Mn_1.5_O_4_-graphene composite electrode recover to the original values, indicating that large current density and rapid lithiation/delithiation did not cause any permanent damage to the crystal structure. However, after the bare LiNi_0.5_Mn_1.5_O_4_ nanorod electrode experienced the high current rate like 10 C, its reversible capacity didn't fully recover to the initial value when the current rate was changed back to 0.1 C. The superior rate performance of the LiNi_0.5_Mn_1.5_O_4_-graphene composite electrode can be attributed to the improved electron transport provided by the graphene conductive matrix. As confirmed by the EIS measurements, the LiNi_0.5_Mn_1.5_O_4_-graphene composite electrode shows much smaller charge transfer resistance compared to the bare LiNi_0.5_Mn_1.5_O_4_ nanorod electrode, indicating the graphene wrapping is beneficial to fast electrode kinetics (Fig. S4, Supporting Information).

## Discussion

Although the conductivity of the ordered LiNi_0.5_Mn_1.5_O_4_ is relatively low, the ordered LiNi_0.5_Mn_1.5_O_4_ nanorod-graphene composite developed in this work exhibited excellent rate capacity as well as good cycling stability. The rate performance of the present ordered LiNi_0.5_Mn_1.5_O_4_-graphene composite is even better than those of the previously reported disordered LiNi_0.5_Mn_1.5_O_4_-grahene composite and disordered LiNi_0.5_Mn_1.5_O_4_-grahene oxide composite^21^,^22^, which can be attributed to its unique hybrid electrode design. First, the one-dimensional nanostructure of ordered LiNi_0.5_Mn_1.5_O_4_ provides short solid diffusion length for lithium ions. This, along with fast electron transport supplied by the highly conductive graphene matrix, endows the hybrid electrode with fast lithiation and delithiation capability. Second, the ordered LiNi_0.5_Mn_1.5_O_4_ nanorods with *P*4_3_32 symmetry contain negligible Mn^3+^ ions, which minimizes Mn disproportionative dissolution and Jahn-Teller structural distortion, resulting in good structural stability during cycling. Last, the graphene wrapping effectively modify the surface of LiNi_0.5_Mn_1.5_O_4_, which suppresses the side reactions at the electrode/electrolyte interface, resulting in a slow cell polarization growth and excellent cycling stability. Unlike disordered LiNi_0.5_Mn_1.5_O_4_, the ordered LiNi_0.5_Mn_1.5_O_4_ do not have the 4 V voltage plateau, thus making the LiNi_0.5_Mn_1.5_O_4_-grpahene composite more promising for application in high energy and high power lithium-ion batteries.

In summary, a smart hybrid cathode material featuring graphene nanosheets wrapped one-dimensional ordered LiNi_0.5_Mn_1.5_O_4_ nanorods has been developed by a facile method combining oxide template synthesis and chemical mixing. The structural characterization confirmed the as-prepared LiNi_0.5_Mn_1.5_O_4_ nanorods possess a ordered spinel structure with *P*4_3_32 symmetry. The LiNi_0.5_Mn_1.5_O_4_-graphene composite electrode exhibited excellent rate capability, delivering reversible capacities of 122.4 and 81.2 mAh g^−1^ at 0.1 and 10 C, respectively. In addition to the fast charge and discharge capability, the LiNi_0.5_Mn_1.5_O_4_-graphene composite electrode also revealed promising cycling stability, with 94% capacity retained after 200 cycles. In comparison with the bare LiNi_0.5_Mn_1.5_O_4_ nanorod electrode, the composite electrode showed greatly improved electrochemical performance, which can be ascribed to the smart hybrid electrode design, enabling both fast charge transport and good interface stability. The superior electrochemical performance and the facile synthesis procedure make the present LiNi_0.5_Mn_1.5_O_4_-graphene composite promising as cathode for high energy lithium-ion batteries.

## Methods

### Preparation β-MnO_2_ nanowires and ordered LiNi_0.5_Mn_1.5_O_4_ nanorods

β-MnO_2_ nanowires were prepared by a modified hydrothermal method according to the literature^34^. In a typical synthesis, 10 g KNO_3_ were added into a solution containing 20 ml 50 wt% MnNO_3_ and 20 ml deionized water to get a supersaturated system. Then the well mixed solution was transferred into a 50 mL Teflon-lined stainless steel autoclave and heated at 140 °C for 14 h. After cooling down to room temperature, the precipitated product was filtered and washed sequentially with deionized water and ethanol for three times. The obtained β-MnO_2_ nanowires were then dried at 80 °C for 6 h in air for further usage. The ordered spinel LiNi_0.5_Mn_1.5_O_4_ nanorods were then synthesized by using the β-MnO_2_ nanowires as the template. Briefly, stoichiometric amounts of Ni(CH_3_COO)_2_, LiOH·H_2_O, and β-MnO_2_ nanowires were homogeneously dispersed in high purity ethanol and stirred for 24 h. The obtained precursor was dried at 60 °C for 3 h and ground in a mortar for 1 h. After that, the mixture was first preheated at 300 °C for 5 h and then calcined at 700 °C for 10 h in air to obtain the ordered LiNi_0.5_Mn_1.5_O_4_ nanorods.

### Preparation of LiNi_0.5_Mn_1.5_O_4_-graphene composite

The grapnehe nanosheets were prepared from graphite powder in a two-step process, involving the oxidation and/or exfoliation of graphite to graphite oxide by Hummer's method and chemical reduction of graphite oxide to graphene according to literature^35^. To prepare the graphene wrapped LiNi_0.5_Mn_1.5_O_4_ nanorods, 0.05 g graphene and 1 g LiNi_0.5_Mn_1.5_O_4_ nanorods were first mixed in a mortar for 0.5 h by grinding, and then dispersed in 30 ml ethanol by ultrasonication. After that, the dispersion was vigorously stirred at 50 °C for 8 h to achieve a uniform dispersion of LiNi_0.5_Mn_1.5_O_4_ nanorods in the graphene matrix. Finally, the obtained mixture was dried in an oven at 80 °C overnight to obtain LiNi_0.5_Mn_1.5_O_4_-graphene composite.

### Materials Characterization

Structural features of the as-prepared LiNi_0.5_Mn_1.5_O_4_ nanorods and the LiNi_0.5_Mn_1.5_O_4_-graphene composite were characterized with X-ray diffraction (XRD), Raman spectroscopy, and Fourier transform infrared (FTIR) spectroscopy. The XRD patterns were taken by a Shimadzu XRD-6000 X-ray diffractormeter with Cu Kα radiation between 10 and 80°. Raman spectra of different samples were acquired using a Renishaw in Via Reflex Raman microprobe with a 532 nm wavelength incident laser. FTIR spectra of the samples were collected from 800 to 400 cm^−1^ using a Nicolet-670 FTIR spectrometer. To determine the graphene content in the composite, thermogravimetric analysis (TGA) was carried out in the air at a heating rate of 10 °C min^−1^ from 30 to 700 °C using a DTG-60H Shimadzu thermal analyzer. The morphology features of the as-prepared samples were characterized with field-emission scanning electron microscopy (FESEM, JSM-6700F 15 kV), transmission electron microscopy (TEM) and high-resolution transmission electron microscopy (HRTEM, JEOL 2010 200 kV).

### Electrochemical Measurements

To investigate the electrochemical properties, half cells using lithium foil as both counter and reference electrodes were assembled with Lab-made Swagelok cells in an Ar-filled glove box. To prepare the working electrodes, active cathode materials (LiNi_0.5_Mn_1.5_O_4_ nanorods and LiNi_0.5_Mn_1.5_O_4_-graphene composite), acetylene black (Super-P) and polyvinylidene fluoride (PVDF) with a weight ratio of 8:1:1 were mixed with N-methyl-2-pyrrolidinone (NMP) to form a slurry. The obtained slurry was coated onto the Al foils and dried at 120 °C for 2 h to remove the solvent. The electrodes were then pressed and cut into small disks (10 mm in diameter). The small disks were further dried at 80 °C in a vacuum oven for 12 h before battery tests. 1 M LiPF_6_ in ethylene carbonate and diethyl carbonate (EC/DEC, v/v = 1:1) solution was used as the electrolyte and Celgard 2400 membrane was used as the separator. The galvanostatic charge/discharge measurements were carried out on a LAND CT2001A electrochemical workstation with a voltage window between 3.0 and 4.9 V (vs. Li^+^/Li) at different current densities (1 C is 148 mAh g^−1^ for LiNi_0.5_Mn_1.5_O_4_) at room temperature. Cyclic voltammogram (CV) and electrochemical impedance spectroscopy (EIS) measurements were performed using a CHI660D electrochemical workstation. CVs were measured between 3.5 and 5.0 V (vs. Li/Li^+^) at a scan rate of 0.05 mV s^−1^. EIS measurements were carried out in the frequency range between 100 kHz to 0.01 Hz with an AC amplitude of 5 mV at an open circuit potential.

## Additional Information

**How to cite this article**: Tang, X. *et al.* Graphene wrapped ordered LiNi_0.5_Mn_1.5_O_4_ nanorods as promising cathode material for lithium-ion batteries. *Sci. Rep.* **5**, 11958; doi: 10.1038/srep11958 (2015).

## Supplementary Material

Supplementary Information

## Figures and Tables

**Figure 1:**
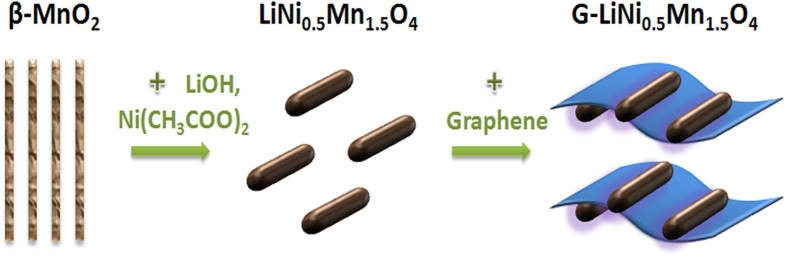
Schematic illustration of the design of the hybrid LiNi_0.5_Mn_1.5_O_4_-graphene electrode material.
