# Supplementary material for: Graphene wrapped ordered LiNi0.5Mn1.5O4 nanorods as promising cathode material for lithium-ion batteries
Source: Sci Rep. 2015 Jul 7;5:11958. doi: 10.1038/srep11958 (PMC4493710; doi:10.1038/srep11958)
Supplement: Supplementary Information [file srep11958-s1.doc]

**Supporting Information**

**Graphene wrapped ordered LiNi_0.5_Mn_1.5_O_4_ nanorods as promising cathode material for lithium-ion batteries**

Xiao Tang^1,2^, S. Savut Jan^1,2^, Yanyan Qian^1,2^, Hui Xia1,2[[1]](#footnote-2)*, Jiangfeng Ni3[[2]](#footnote-3)*, Serguei V. Savilov^4^, Serguei M. Aldoshin^5^

^1^School of Materials Science and Engineering, Nanjing University of Science and Technology, Nanjing 210094, China

^2^Herbert Gleiter Institute of Nanoscience, Nanjing University of Science and Technology, Nanjing 210094, China

^3^College of Physics, Optoelectronics and Energy, Soochow University, Suzhou 215006, China

^4^Department of Chemistry, M. V. Lomonosov Moscow State University, Moscow 119991, Russia

^5^Department of Physical Chemistry Engineering, M. V. Lomonosov Moscow State University, Moscow 119991, Russia


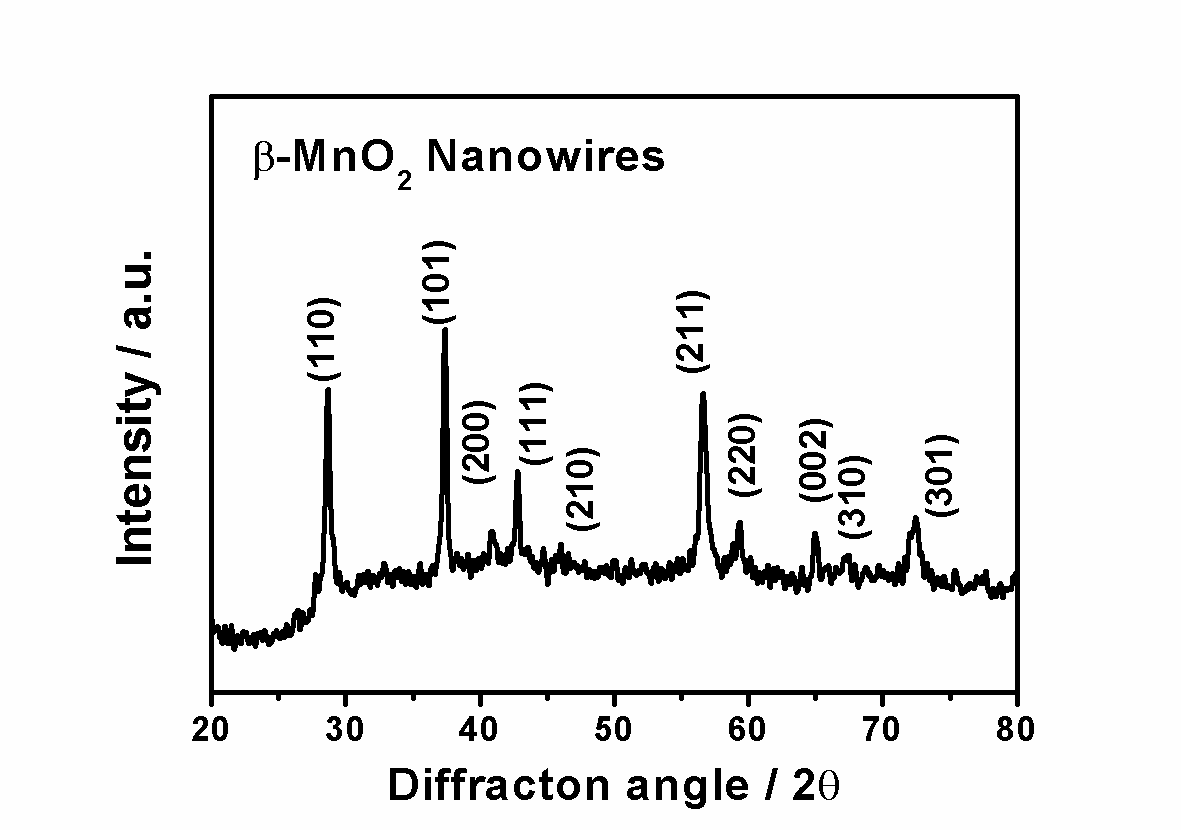


**Figure S1.** XRD pattern of the as-prepared β-MnO_2_ nanowires.


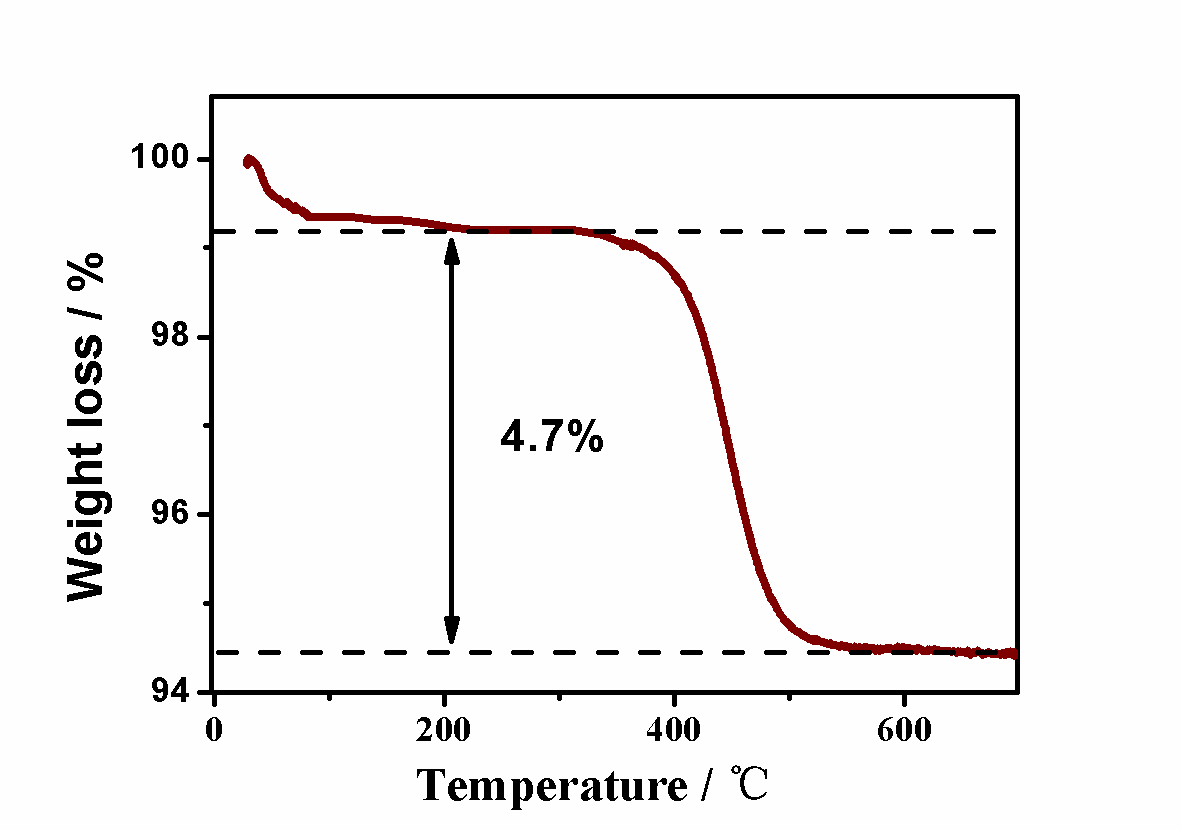


**Figure S2.** TGA curve of the LiNi_0.5_Mn_1.5_O_4_-graphene composite.


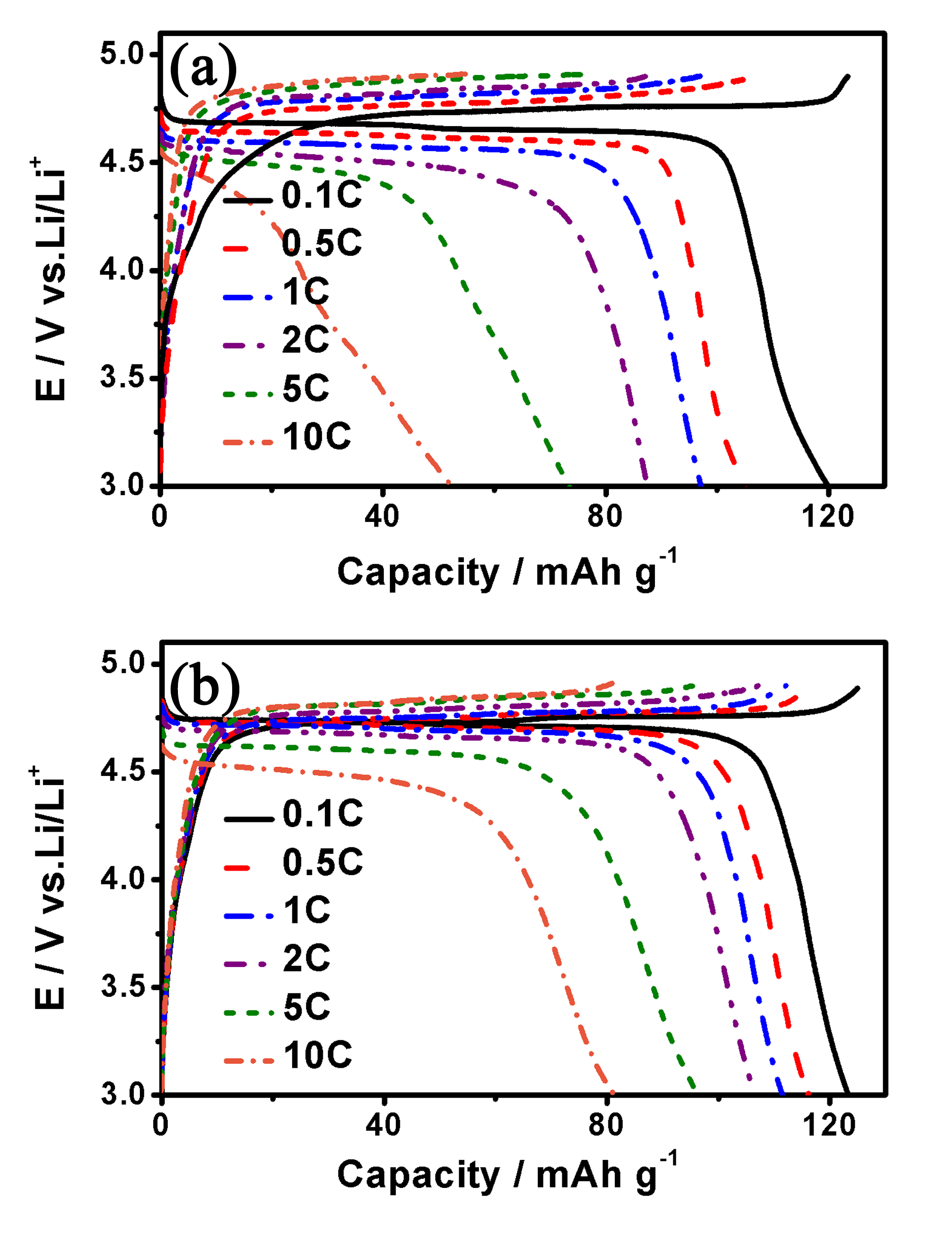


**Figure S3.** Charge/discharge curves of the pristine LiNi_0.5_Mn_1.5_O_4_ nanorod electrode (a) and the LiNi_0.5_Mn_1.5_O_4_/graphene compsoite electrode (b) at differnt current rates.


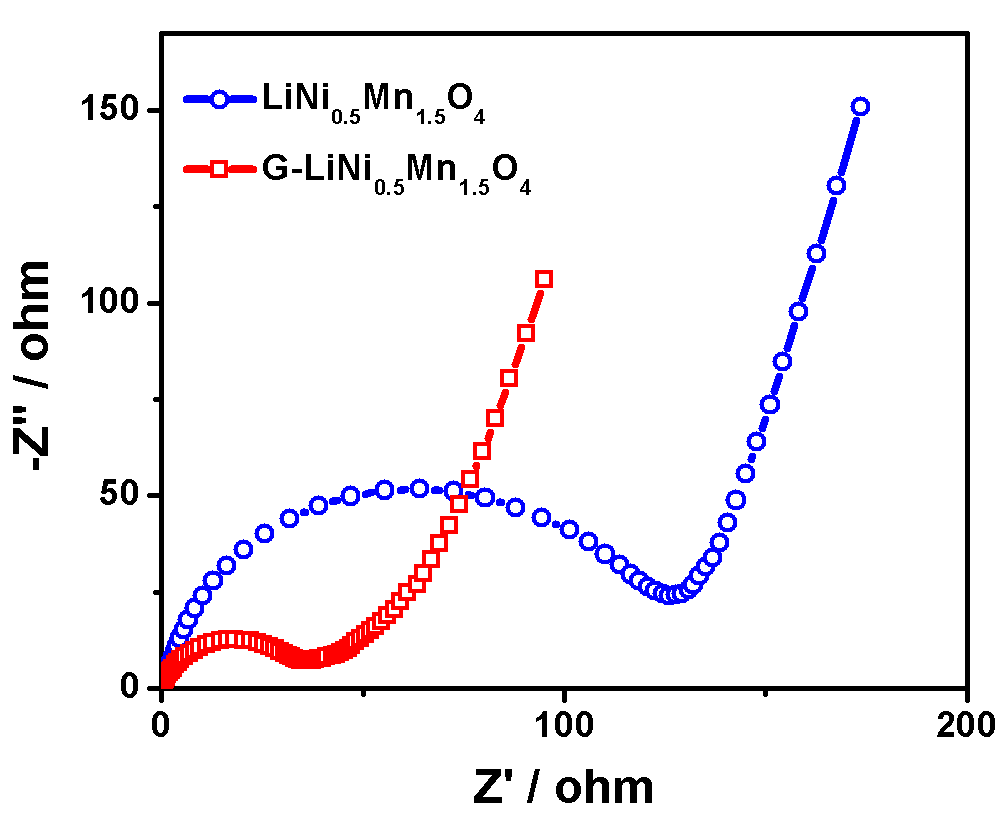


**Figure S4.** EIS spectra of the bare LiNi_0.5_Mn_1.5_O_4_ nanorod electrode and the LiNi_0.5_Mn_1.5_O_4_-graphene composite electrode.

1. ^^ Corresponding author, e-mail: xiahui@njust.edu.cn Tel: (86) 25 84303408 , Fax: (86) 25 84303408 [↑](#footnote-ref-2)
2. ^^ Corresponding author, e-mail: jeffni@suda.edu.cn [↑](#footnote-ref-3)
